# Nanoindentation Response of Monocrystalline Copper via Molecular Dynamics: Anisotropic Edge Effects

**DOI:** 10.3390/mi16050570

**Published:** 2025-05-12

**Authors:** Desong Du, Peng Wu, Huan Liu, Zhengkun Li, Jiubin Tan

**Affiliations:** 1 School of Astronautics, Harbin Institute of Technology, Harbin 150001, China; dudesong@hit.edu.cn; 2 Center of Ultra-Precision Optoelectronic Instrumentation Engineering, Harbin Institute of Technology, Harbin 150001, China; 23s136325@stu.hit.edu.cn (P.W.); jbtan@hit.edu.cn (J.T.); 3 Key Laboratory of Ultra-Precision Intelligent Instrumentation, Ministry of Industry Information Technology, Harbin 150080, China; 4 Key Laboratory of Electrical Quantum Standards for State Market Regulation, National Institute of Metrology, Beijing 100029, China

**Keywords:** nanoindentation, mechanical properties, anisotropic edge effects, molecular dynamics

## Abstract

In the nanoindentation testing of metallic materials, mechanical properties often decrease significantly when the indentation position shifts from the central region to the edge due to edge effects, leading to premature edge failure and potential device malfunctions. In this work, molecular dynamic (MD) simulations were conducted to investigate the anisotropic edge effects of nanoindentation on monocrystalline copper with a specific crystal orientation. The results reveal that changes in indentation position strongly influence surface collapse and lateral pile-up behaviors. Notably, edge positions resulted in significant reductions in indentation force and hardness, accompanied by pronounced anisotropy in nanoindentation hardness. Additionally, distinct von Mises stress distributions were observed at different indentation positions, highlighting the crystallographic orientation’s role in modulating edge effects. This study provides new insights into the atomic-scale mechanisms underlying edge effects in metallic materials and their anisotropic characteristics.

## 1. Introduction

**Figure 1:**
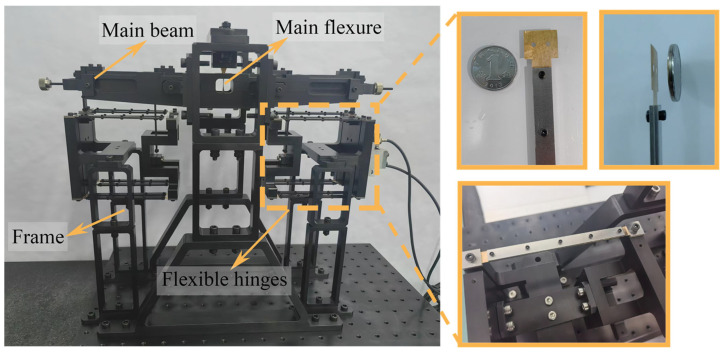
Application of copper as a flexible material in precision instruments.

Copper, owing to its exceptional flexibility and elastic recovery, is extensively utilized in the design and manufacture of flexible mechanisms, as demonstrated by its widespread application in precision testing instruments [1,2] and precision guiding systems [3,4], as shown in Figure 1. Concurrently, the plastic deformation characteristics of copper play a critical role in the overall performance of these flexible mechanisms. Consequently, a comprehensive investigation into the plastic deformation mechanisms of copper not only aids in exploring strategies to enhance material properties while retaining its inherent flexible guiding capabilities but also provides crucial insights for evaluating the mechanical performance of copper when fabricated into fine wires or other flexible components [5,6,7,8,9]. Presently, extensive research on the mechanical properties of copper is being conducted globally, with nanoindentation and similar techniques commonly employed to analyze the mechanical behavior of nanoscale metals [10,11,12,13,14].

Copper thin films are usually used as research subjects in nanoscale indentation experiments due to their excellent physical and mechanical properties. Shi et al. used MD simulations to study the effect of water thin films on the plastic deformation of Cu thin films during nanoscale indentation at different loading rates and indentation speeds [15]. They found that as the thickness of the water thin film and the indentation speed/loading rate increased, the maximum load increased, but the maximum penetration depth decreased. Hong et al. assessed the elastic modulus in both the vertical and parallel directions to the surface of Cu thin films using nanoindentation tests [16]. They discovered that nanoindentation techniques can quantitatively characterize the anisotropic elastic modulus of textured metal thin films. Lu et al. employed a quasi-continuous multiscale approach to investigate the position effect of cylindrical indenters in nanoscale indentation on Cu thin films [17]. They found that the initial plastic deformation is significantly influenced by the indenter’s position during nanoindentation. Wang et al. conducted constant load creep experiments to study nanoscale indentation creep in polycrystalline Cu thin films [18]. They observed that the penetration depth is time-dependent and exhibits a sawtooth pattern at low loads. Peng et al. performed nanoindentation tests on the Cu thin film substrate to study the reinforcement mechanism of graphene coatings [19]. They found that covering the substrate with a graphene coating can enhance its load-bearing capacity.

In addition, Sun et al. investigated the creep behavior of nanocrystalline Cu with an average grain size of 25 nanometers using room temperature nanoindentation tests [20]. The experimental results showed that as the creep stress increased, the activation volume first increased and then decreased, and the strain rate sensitivity first decreased and then increased. Liu et al. conducted nanoindentation creep tests on nanocrystalline Cu at room temperature [21]. They found that the stress exponent for nanoindentation creep at room temperature was very high, indicating a different creep mechanism from power law creep. Tan et al. conducted static atomistic simulations to study the influence of vacancies on Cu (1 1 1) nanoindentation [22]. The study found that the presence of vacancies could reduce the nanohardness by approximately 10% to 15%. Liu et al. evaluated the creep behavior of nanocrystalline copper at room temperature after reducing the stress using nanoindentation techniques [23]. The research results showed that under moderate stress reduction conditions, although dislocation slip ceased, processes mediated by grain boundaries often dominated. Chen et al. used nanoindentation to study the critical shear stress for plastic initiation in magnetron-sputtered nanocrystalline Cu thin films with an average grain size of 14 nanometers [24]. They found a high critical shear stress during the plastic initiation stage in Cu samples with an average grain size of 14 nanometers. Liu et al. conducted nanoindentation experiments on monocrystalline Cu to consider the formation of layer fault tetrahedra [25]. They proposed a new mechanism to explain the formation of stacking fault tetrahedra (SFT) in bulk monocrystalline Cu with pre-existing parallel coherent twin boundaries during the plastic deformation process.

With the development of micro-electro-mechanical system (MEMS) and nano-electro-mechanical system (NEMS) devices, the size of metal devices is continuously shrinking, and the proportion of the device edge region is increasing. Due to surface effects, there is a significant difference in the mechanical properties of nanoscale indentations at the material’s edge compared to its interior. This characteristic often leads to the failure of the material’s edge under the same operating conditions, also known as edge effect, resulting in device failure. Therefore, understanding the edge effects of monocrystalline Cu can help predict the device’s lifespan. Despite many studies on the microstructure and mechanical properties of Cu and Cu alloys [26,27,28,29,30,31,32,33], there is still a lack of research on Cu edge effects during the nanoscale indentation process.

In this work, the MD simulations were employed to investigate the edge effects of nanoscale indentation on single crystal copper. The MD simulation results indicate that the edge effects have a significant impact on the mechanical properties and deformation processes of the material, leading to notable changes in surface collapse, side pile-up, indentation force, indentation hardness, stress distribution, and so on. This work can be used to gain an atomic-level understanding of the edge effects in nanoscale indentation of metals.

## 2. Materials and Methods

**Figure 2:**
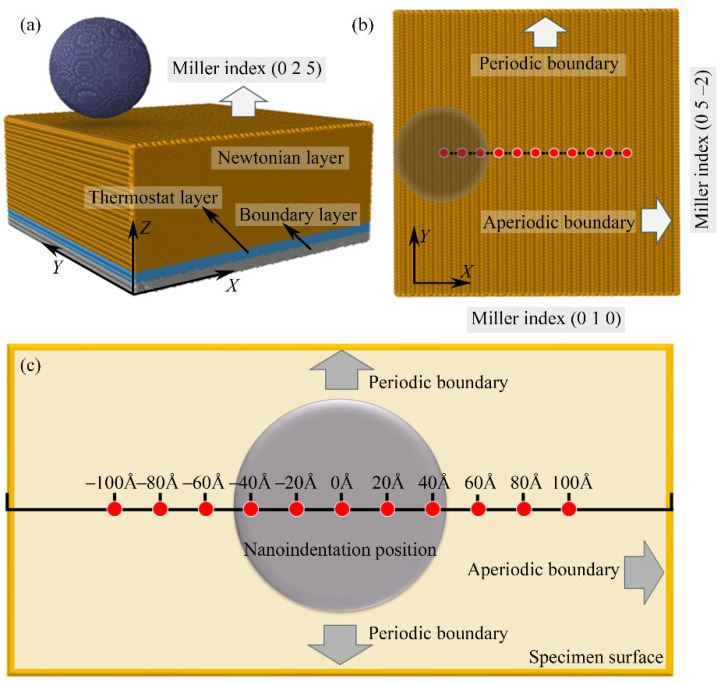
(**a**) The MD model for nanoindentation edge effect. (**b**) The Miller index of the MD model. (**c**) The schematic diagram of the indentation positions.

The molecular dynamic (MD) bending simulation model of the nanoindentation, as shown in Figure 2, uses single-crystal copper (Cu) with a lattice constant of 3.615 Å. The MD model dimensions are 100 Å × 500 Å × 250 Å, containing approximately 1,066,464 atoms. The crystal orientations along the *X*-, *Y*-, and *Z*-axis of the MD model are [1 0 0], [0 1 0], and [0 0 1], respectively. The nanoindentation simulation model is divided into three regions: the Newton layer, thermostat layer, and fixed layer. In the Newton layer, atoms undergo primary deformation, following Newton’s laws using the velocity–Verlet method [31]. The thermostat layer maintains a constant temperature of 293 K, regulated by the Berendsen thermostat [32]. Atoms in the fixed layer retain initial parameters to support the system. To minimize size effects, periodic boundary conditions (PBCs) are applied in the *X*-, *Y*-, and *Z*-axis.

The MD simulation was conducted to investigate the nanoindentation edge effect of monocrystalline copper. As depicted in Figure 2, the MD model employed for the nanoindentation study comprised a diamond indenter and a monocrystalline specimen. The diamond indenter possessed a radius of 5 nm and consisted of 92,061 C atoms. The monocrystalline copper specimen had dimensions of 30 nm × 30 nm × 15 nm, with 1,141,914 Cu atoms. The specimen was stratified into three distinct layers: the Newtonian layer, the thermostat layer, and the boundary layer. The boundary layer provided structural support for the entire nanoindentation system, whereas the thermostat layer facilitated the dissipation of heat generated during the indentation process. Within the Newtonian layer, the motion of atoms under the influence of the diamond indenter adhered to Newton’s laws of motion.

To capture the influence of edge effects, a periodic boundary condition was applied along the *Y*-direction, while the *X*-direction boundary was characterized as aperiodic, enabling the study of variations at different positions along the edge. As illustrated in Figure 2b, the Miller indices for the *X*-direction, *Y*-direction, and *Z*-direction were (0 1 0), (0 5 −2), and (0 2 5), respectively. The (0 2 5) crystal orientation was deliberately selected to introduce crystallographic inconsistency along the *X*-direction [34]. This unique orientation induces structural asymmetry at the edges of the specimen, providing an ideal condition to investigate how edge effects differ on opposing sides. Such asymmetry is critical for examining the anisotropic nature of edge effects, as it highlights directional dependencies in material responses under nanoindentation. Furthermore, the indentation positions were uniformly distributed at 2 nm intervals along the *X*-direction, facilitating a systematic investigation of the edge region’s influence on nanoindentation behavior. As shown in Figure 2c, the designated indentation position *L* was defined as the deviation distance along the positive *X*-direction from the center of the monocrystalline Cu specimen, ranging from –100 Å to 100 Å in 20 Å increments. This approach ensured the comprehensive coverage of both the central and edge regions, enabling a detailed analysis of position-dependent responses. To quantify the size variation in the material, the change in the size of the copper workpiece is described based on the change in its basic size in this simulation.

**Figure 3:**
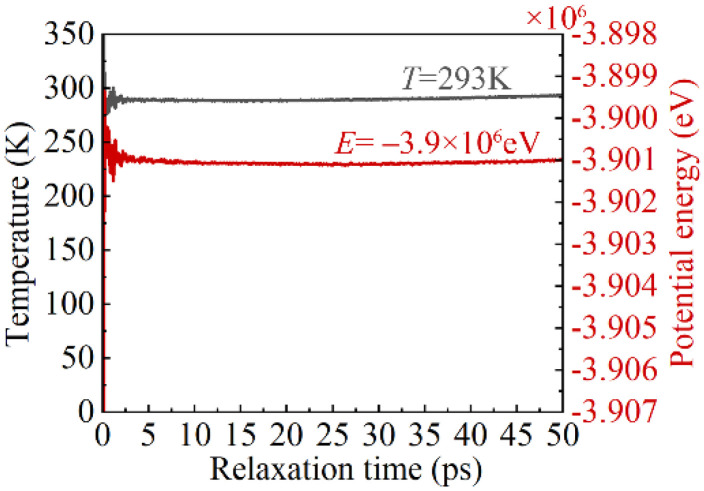
The variations in the temperature and total potential energy of the specimen system during the relaxation process.

The MD simulation process mainly consists of two consecutive stages, the relaxation stage and the nano-indentation stage. In the relaxation stage, the MD system first undergoes energy minimization using the conjugate gradient method. Subsequently, a relaxation process is carried out within the NPT ensemble at a relaxation temperature of 293 K and a pressure of 1 atmosphere for a relaxation time of 50 ps, leading to the attainment of equilibrium for the MD model. The variations in the temperature and total potential energy of the system during the relaxation process are shown in Figure 3. In the nanoindentation stage, nanoindentation simulation was performed within the NVE ensemble with a loading velocity of 50 m/s and a nanoindentation depth of 3.5 nm. The MD simulation parameters are provided in Table 1.

The interactions of Cu atoms were described by the EAM potential function [35,36]. The total energy *E_i_* of an atom *i* is given as

(1) $$E_{i}=F_{\alpha }\left(\sum_{j\neq i}\rho_{\beta }\left(r_{ij}\right)\right)+\frac{1}{2}\sum_{j\neq i}\phi_{\alpha \beta }\left(r_{ij}\right),$$

where *F* is the embedding energy which is a function of the atomic electron density *ρ*, *Φ* is a pair potential interaction, and *α* and *β* are the element types of atoms *i* and *j*. The multi-body nature of the EAM potential is a result of the embedding energy term. Both summations in the formula are above all neighbors *i* of atom *j* within the cutoff distance.

The Morse potential was used to describe the interactions of C-Cu, as shown in Equation (2).

(2) $$E=D_{0}\left[e^{-2\alpha \left(r-r_{0}\right)}-2e^{-\alpha \left(r-r_{0}\right)}\right]\begin{array}{cc} & r<r_{c} \end{array},$$

where *E* is the potential energy; *r* is the distance between the C and Cu atoms; *D*_0_ is the binding energy; *α* is the elasticity modulus; *r*_0_ is the atomic spacing; and *r_c_* is the cutoff radius. Morse potential parameters are shown in Table 2 [37].

All the MD simulations were conducted using the open-access software Large-scale Atomic/Molecular Massively Parallel Simulator (LAMMPS) (https://www.lammps.org/). The dislocations were identified and quantified with the help of the dislocation extraction algorithm (DXA) using the software OVITO with the version number of 3.12.2 [38,39]. The von Mises stress of atoms was calculated based on the six stress vectors obtained from the MD simulations, as shown in Equation (3).

(3) $$\sigma_{\mathrm{VM}}=\sqrt{{\left(\sigma_{1}-\sigma_{2}\right)}^{2}+{\left(\sigma_{2}-\sigma_{3}\right)}^{2}+{\left(\sigma_{3}-\sigma_{1}\right)}^{2}+6(\tau_{12}^{2}+\tau_{23}^{2}+\tau_{31}^{2})},$$

In order to show the indentation performance of the material more clearly, we calculated the indentation hardness of the workpiece according to the load and the indentation depth. The indentation hardness *H* is defined as the average pressure value below the indentation, as shown in Equation (4).

(4) $$H=\frac{F_{z}}{A_{c}}$$

where *F*_z_ is the force along the *Z*-direction during the indentation and *A*_c_ is the area in the *X*-*Y* plane of the contact part of the spherical indenter with the specimen, as shown in Equation (5).

(5) $$A_{c}=\pi (R^{2}-{\left(R-d\right)}^{2})$$

where *R* is the radius of the spherical indenter and *d* is the indentation depth.

For the nanoindentation data of load indentation depth, we used a Savitzky–Golay filter to fit a local polynomial, which can smooth out noise while avoiding the excessive passivation of the abrupt signal by the moving average method, thus preserving the amplitude and slope information of the load drop point. Choosing a third-order polynomial which is an excessively high polynomial order, can introduce overfitting oscillations, while an excessively low polynomial order can result in insufficient data smoothness. At the same time, a size 20 window should be selected to ensure that local trend fitting does not mask physical mechanisms.

## 3. Results

In this study, we focus exclusively on the influence of edge effects on the nanoindentation behavior of monocrystalline Cu. To isolate the edge effect, all simulations were performed using a diamond indenter with a fixed radius of 5 nm. While the indenter size effect has been extensively studied in the literature [40,41,42], it is not the primary focus of this work. By maintaining a consistent indenter size, we ensure that the observed variations in load–displacement curves and dislocation evolution are solely attributable to the edge effect. This approach allows us to provide a clear and focused analysis of the edge region’s influence on nanoindentation behavior, without the confounding effects of variable indenter sizes.

### 3.1. The Surface Sink-In and Side Squeeze-Out

**Figure 4:**
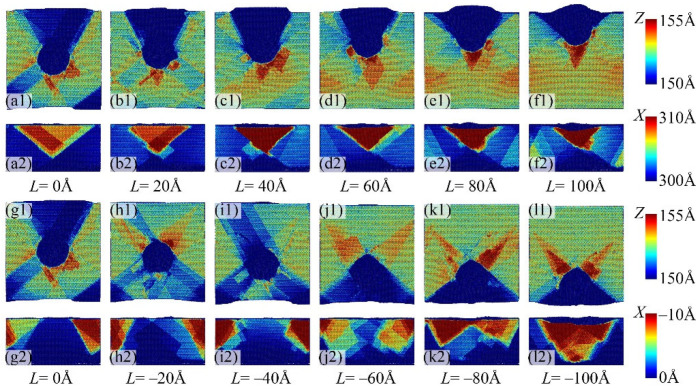
The surface sink-in and side squeeze-out under different indentation positions. The *L* values of (**a**–**l**) are 0, 20, 40, 60, 80, 100, 0, −20, −40, −60, −80, −100 Å, respectively.

As shown in Figure 4, it can be observed that the surface and lateral topographical characteristics undergo significant changes as the indentation position *L* increases. When *L* = −100 Å, a distinct isosceles triangle collapse with a bottom angle of 35° is observed on the *X*-*Y* side of the specimen. Simultaneously, on the side adjacent to the indenter, there forms an isosceles triangle pile-up with a bottom angle of 60°. When *L* = −80 Å, a more extensive yet shallower isosceles triangle collapse is visible on the monocrystalline copper surface, accompanied by the emergence of irregular material pile-ups near the indenter. Conversely, when *L* = −60 Å, a non-triangular surface collapse appears on the workpiece’s surface. For *L* = −40 Å and *L* = −20 Å, noticeable surface collapses and side pile-ups are absent during the nanoindentation procedure. For *L* = 0 Å, an isosceles triangle surface collapse with a bottom angle of 60° is observed on the specimen’s surface, while an isosceles triangle pile-up with a bottom angle of 35° appears on the *X* = 0 nm plane. Similarly, for *L* from 20 Å to 100 Å, the regions containing triangle surface collapses and side pile-ups decrease as the indentation positions progress, accompanied by an increase in the height of side pile-ups.

In addition, it was observed that the surface sink-in observed on the upper surface of the specimen mainly consists of two types. When *L* ≥ 0 Å, the surface sink-in forms an isosceles triangle with a vertex angle of 35°. When *L* < 0 Å, side squeeze-out occurs in the form of an isosceles triangle with a vertex angle of 60°. Three types of side squeeze-out are observed on the side of the specimen. When *L* > 0 Å, the side squeeze-out on the *Y* = 300 Å surface appears as a right-angled triangle. There is no significant side squeeze-out on the *Y* = 0 Å surface. When −80 Å ≤ *L* ≤ 0 Å, significant side squeeze-out is observed at the upper left and upper right corners of the *Y* = 0 Å surface, and when *L* = −100 Å, the side squeeze-out on the *Y* = 0 Å surface changes into an inverted triangle. To explain these observations at the atomic scale, this work employs slip systems-related theories.

**Figure 5:**
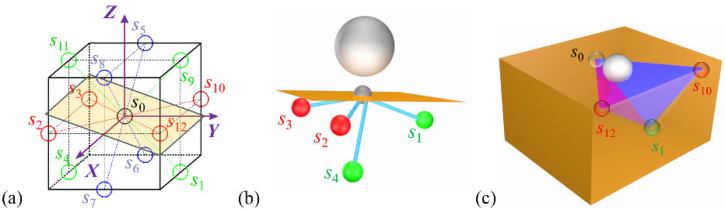
Mechanisms of plastic deformation of materials in indentation edge effects. (**a**) The relative position of the workpiece surface and the [1 1 0] slip direction; (**b**) the activated [1 1 0] slip directions during the indentation process of (0 2 5) surface monocrystalline copper; (**c**) the two (1 1 1) slip planes corresponding to the [1 1 0] slip direction *s*_i_.

The differences in surface sink-in and side squeeze-out phenomena induced by various indentation edge effects can be explained by the atomic-scale motion of FCC crystalline [1 1 0] (1 1 1) slip systems. As shown in Figure 5, the monocrystalline copper specimen comprises twelve [1 1 0] slip directions, labeled *s*_1_ to *s*_12_. The upper surface of the specimen is of (0 2 5) crystalline structure, and under the influence of the diamond indenter, slip directions *s*_1_ to *s*_4_ become active, as depicted in Figure 5b. Additionally, for each [1 1 0] slip direction, there are two (1 1 1) slip planes with normal vectors perpendicular to the corresponding [1 1 0] slip direction, which dominate in terms of the number of slip atoms. Therefore, the primary slip systems of monocrystalline copper during the nanoindentation process are exemplified in Figure 5c.

**Figure 6:**
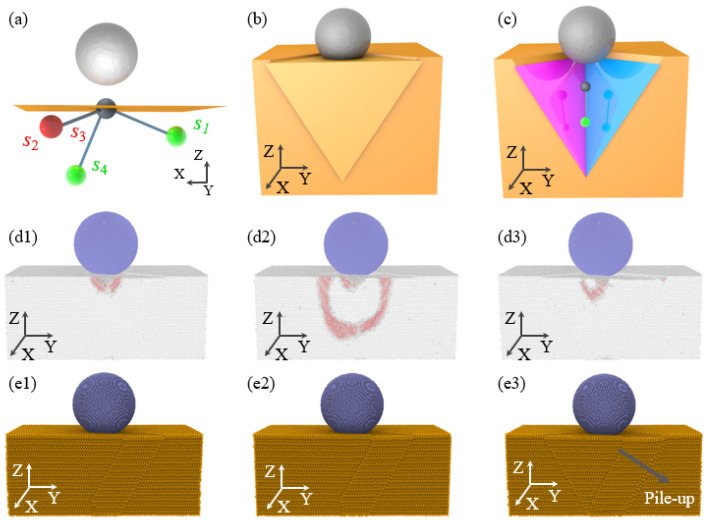
Schematic diagram of the formation mechanism and MD simulation results for surface sink-in and side pile-up under indentation position 50. (**a**–**c**) Schematic diagram of the formation mechanism for surface sink-in and side pile-up; (**d1**–**d3**) generation, propagation, and disappearance of HCP dislocation structures obtained by MD simulation; and (**e1**–**e3**) MD results of surface sink-in and side pile-up corresponding to (**d1**–**d3**).

According to the slip model shown in Figure 5, when *L* = −100 Å, surface sink-in and side squeeze-out phenomena are primarily controlled by the activated slip direction *s*_1_. When single crystal atoms slip along the (1 1 0) plane with slip direction *s*_1_, the specimen’s edge forms an isosceles triangle surface sink-in with a base angle of 35° and an isosceles triangle side squeeze-out with a base angle of 65°, as shown in Figure 6a–c. Figure 5(d1)–(d3) elucidate the motion of dislocations within the hexagonal close-packed (HCP) crystal structure during nanoscale indentation. It was observed that an HCP dislocation is formed at the bottom of the diamond indenter, as shown in Figure 6(d1), and subsequently, the HCP dislocation moves towards the bottom of the single crystal copper specimen, as shown in Figure 6(d2), ultimately disappearing at the specimen’s bottom, as shown in Figure 6(d3). This process of dislocation generation, propagation, and annihilation repeats continuously, eventually resulting in surface sink-in and side squeeze-out at the specimen’s edge. It should be emphasized that the HCP dislocation shown in Figure 6(d2) corresponds to the slip plane shown in Figure 6c.

**Figure 7:**
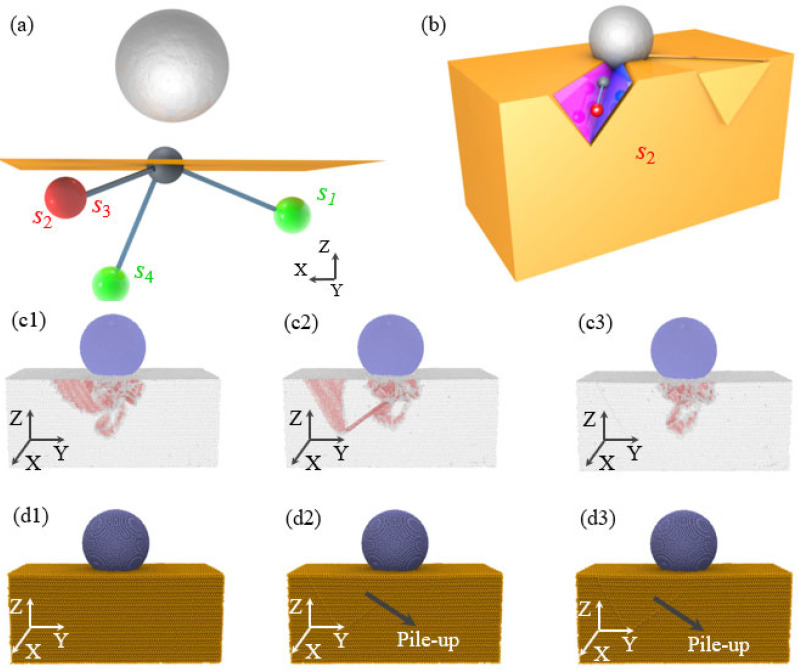
Schematic diagram of the formation mechanism and MD simulation results for surface sink-in and side pile-up under indentation positions 70 Å and 90 Å. (**a**,**b**) Schematic diagram of the formation mechanism for surface sink-in and side pile-up; (**c1**–**c3**) Generation, propagation, and disappearance of HCP dislocation structures obtained by MD simulation; and (**d1**–**d3**) MD results of surface sink-in and side pile-up corresponding to (**c1**–**c3**).

In the case of *L* = −80 Å, during the nanoindentation process, slip directions *s*_2_ and *s*_3_ are activated. When monocrystalline copper atoms undergo slip along the (1 1 1) crystal planes corresponding to *s*_2_ and *s*_3_, as shown in Figure 7a, the resulting surface deformation and accumulation are consistent with the results of molecular dynamics simulations shown in Figure 4(k1,k2). Furthermore, Figure 6(c1–c3) demonstrate the motion of HCP dislocations within the specimen under the action of the diamond indenter. It is observed that HCP dislocations are generated and move on the (1 1 1) slip planes, leading to the formation of triangular accumulations on one side of the monocrystalline copper sample, as shown in Figure 6(d1–d3). Similarly, at the indentation positions of *L* = −60 Å and *L* = −80 Å, due to the activation of slip directions *s*_2_ and *s*_3_, surface deformation and lateral accumulation also occur.

**Figure 8:**
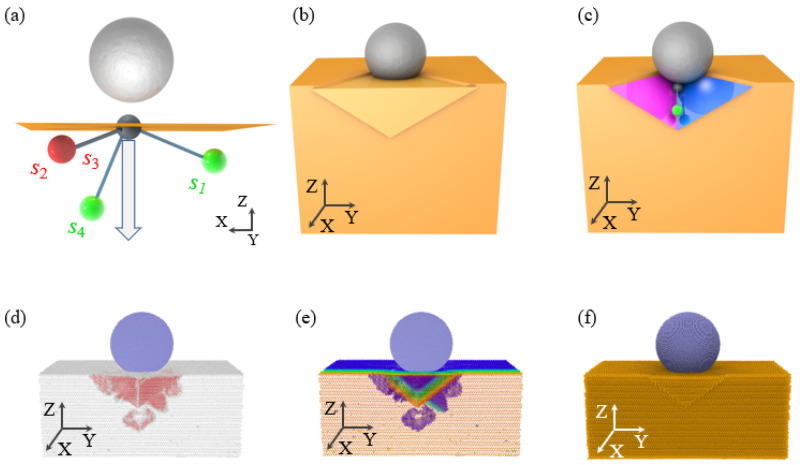
Schematic diagram of the formation mechanism and MD simulation results for surface sink-in and side pile-up when the indentation position is large than 150 Å. (**a**–**c**) Schematic diagram of the formation mechanism for surface sink-in and side pile-up; (**d**–**f**) MD simulation results for the distribution of HCP dislocation structures, surface sink-in, and side pile-up corresponding, where the HCP atoms is colored by *X* coordinate.

When *L* > 0, the slip direction *s*_4_ of the specimen is activated, exerting a dominant influence on surface sink-in and side squeeze-out phenomena, as shown in Figure 8a–c. Figure 8d,e depict the distribution of HCP dislocations in single crystal copper specimens, corresponding to the slip plane in Figure 8c. Furthermore, the (1 0 0) slip plane associated with the slip direction *s*_4_ is closely linked to the indentation position. According to our previous research, the (1 0 0) slip plane aligned with the slip direction *s*_4_ should be tangential to the edge of the diamond indenter. Additionally, as *L* increases, the extent of surface sink-in and side squeeze-out regions decreases.

**Figure 9:**
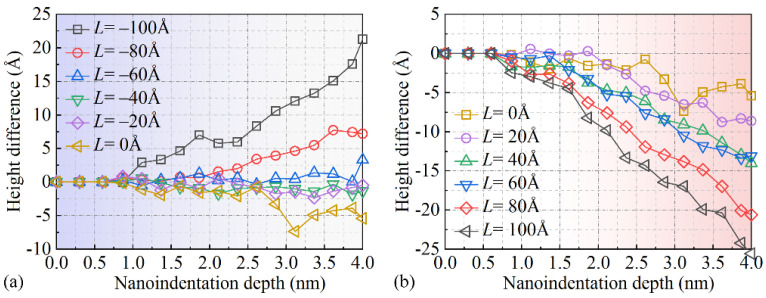
The variations in height difference caused by surface sink-in with respect to indentation depth under different indentation positions. (**a**) Height difference curve when *L* from -100 to 0 Å. (**b**) Height difference curve when *L* from 0 to 100 Å.

Figure 9 illustrates the variation in height along the *Z*-direction of surface sink-in at different nanoscale indentation positions with respect to the change in nanoscale indentation depth. It can be seen that when the indentation depth is less than 0.5 nm, there is no significant plastic deformation during the nanoindentation process of monocrystalline copper. Therefore, at an indentation depth of 0.5 nm, there is no noticeable surface sink-in phenomenon in monocrystalline copper. As the indentation depth continues to increase, the height difference caused by surface sink-in becomes significantly more pronounced. As shown in Figure 9a, it can be seen that when *L* = −80 Å and *L* = −100 Å, the height difference in surface sink-in on both sides of the diamond indenter increases to 1.5 nm and 0.5 nm, respectively, when the indentation depth reaches 3.5 nm. This difference in subsurface sinking is primarily attributed to the activation of different [1 1 0] slip directions during the nanoindentation process, as shown in Figure 5 and Figure 6. In contrast, when *L* from −20 Å to −80 Å, the performance of surface sink-in is not significant, indicating that the edge effects related to surface sink-in are weaker at these indentation positions. It is worth noting that when *L* > 0 Å, the height difference in surface sink-in generated during the nanoindentation process significantly increases with increasing *L*. This is because the activated slip direction in the monocrystalline copper during this process is *s*_4_. Thus, it can be observed that monocrystalline copper nanoindentation exhibits anisotropic behavior in terms of surface sink-in when *L* > 0 Å and *L* < 0 Å, as well as different edge effects.

### 3.2. The Distribution of Von Mises Stress

**Figure 10:**
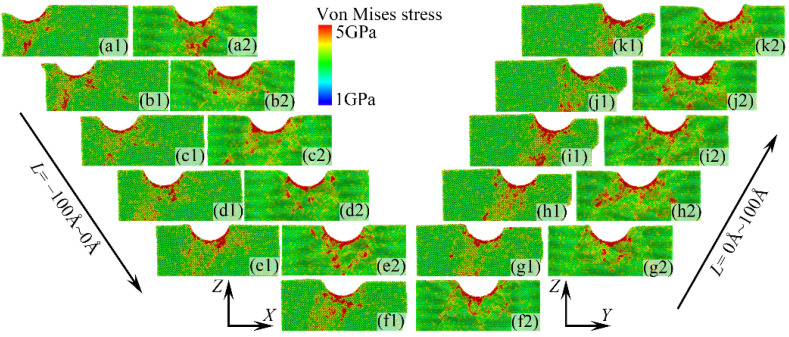
The von Mises stress distribution of the specimen in *X*-*Z* plane (**a1**–**kl**) and *Y*-*Z* plane (**a2**–**k2**) under different indentation positions.

Figure 10 displays the von Mises stress distribution within the *X*-*Z* plane at different indentation positions. When *L* = −80 Å and *L* = −100 Å, there is a higher von Mises stress near the diamond indenter, but no significant von Mises stress distribution is observed within the specimen. This is mainly because a large number of atoms move toward the edges of the specimen, relieving the von Mises stress inside it. Conversely, it was obeserved that when –60 Å ≤ *L* ≤ 80 Å, the von Mises stress is transmitted from the bottom of the diamond indenter into the specimen along the sliding direction *s*_1_, as shown in Figure 7a. Furthermore, it was observed that the range of von Mises stress propagation peaks at *L* = −20 Å. As the indenter approaches the side boundaries of the specimen, the area of von Mises stress distribution gradually decreases due to the influence of the *s*_4_ sliding direction on the atomic motion within the specimen. Additionally, when *L* = 100 Å, similar to the situation when *L* = −80 Å and *L* = −100 Å, the higher von Mises stress distribution is still primarily concentrated in the bottom region of the indenter.

Figure 10 also shows the von Mises stress distribution within the *Y*-*Z* plane of the specimen at different indentation positions. It can be observed that the indentation position has a minimal impact on the von Mises stress distribution within the *Y*-*Z* plane. The main von Mises stress is concentrated in the bottom region of the indenter, and its propagation range into the specimen is relatively small. This is primarily due to the lack of [1 1 0] slip directions within the *Y*-*Z* plane. As indicated by the slip directions labeled *s*_1_–*s*_4_ in Figure 5a, these slip directions do not align with the *Y*-*Z* plane when performing nanoindentation on the (0 2 5) crystal plane of monocrystalline copper. Consequently, the range of propagation within the specimen is limited.

**Figure 11:**
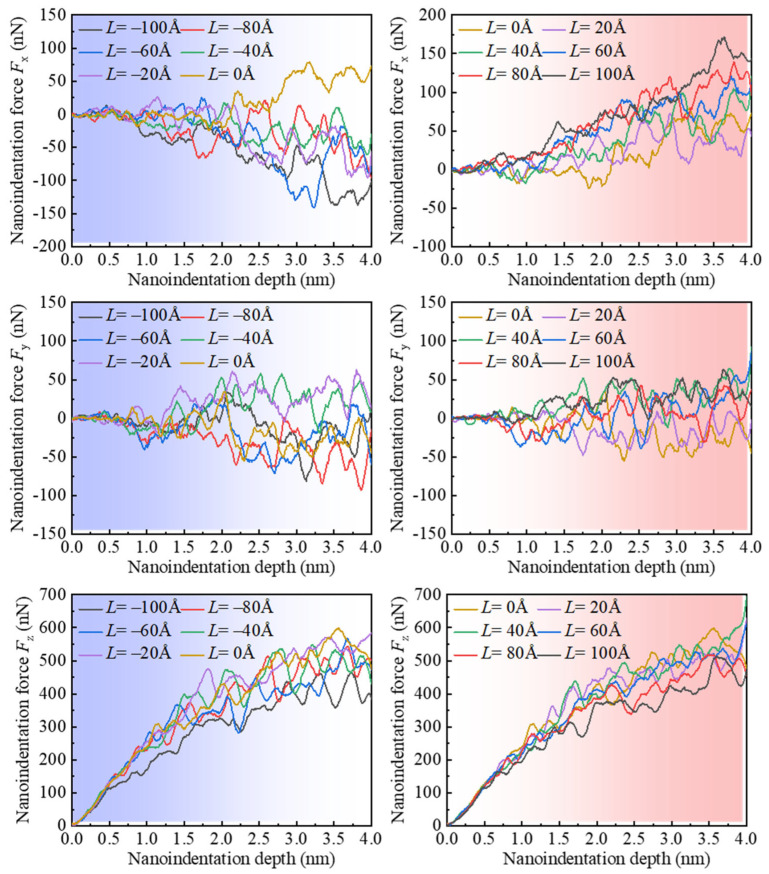
The change in indentation force in *X*-, *Y*-, and *Z*-directions for different indentation positions.

Figure 11 presents the variation in indentation force along different directions with respect to the indentation depth for various indentation positions, denoted by *L*. As shown in Figure 10(a1,a2,b1,b2), when the indentation depth is less than 0.5 nm, the monocrystalline copper specimen primarily undergoes elastic deformation, and *F*_x_ exhibits fluctuations with an average value of approximately 0 nN. As the indentation depth increases, for *L* ≤ −20 Å, the indentation force *F*_x_ tends to be in the negative direction of the *X*-axis. Conversely, for *L* ≥ 0 Å, the indentation force *F*_x_ tends to be greater than 0. Furthermore, it can be observed that the phenomenon of the unbalanced loading of the indentation force *F*_x_ becomes more pronounced when the indentation position approaches the edge of the specimen, especially for *L* ≥ 0 Å. In contrast, there is no significant increase or decrease in the indentation force *F*_y_ with changes in the indentation position. Only with increasing indentation depth does the fluctuation of the indentation force *F*_y_ become more pronounced. Additionally, when the indentation depth is less than 0.5 nm, the variation in the indentation force *F*_z_ is similar for different indentation positions. However, with an increase in indentation depth, when the indentation position is close to the edge of the specimen, the indentation force *F*_z_ undergoes a significant decrease.

**Figure 12:**
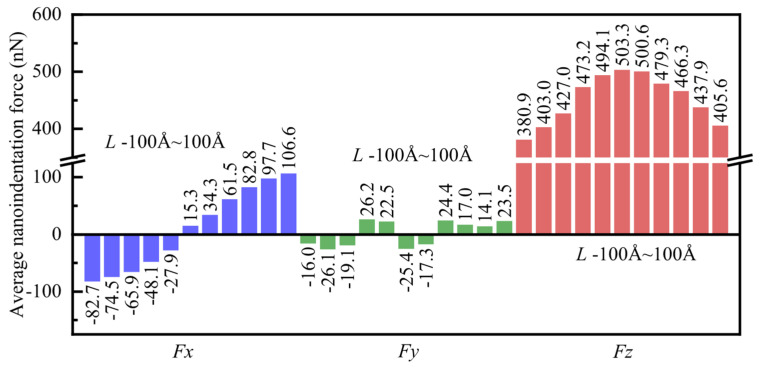
The average nanoindentation force in *X*-, *Y*-, and *Z*-directions for different indentation positions.

The average nanoindentation force in *X*-, *Y*-, and *Z*-directions for different indentation positions is depicted in Figure 12. The range of statistically averaged nanoindentation forces corresponds to indentation depths from 2 nm to 4 nm. It was observed that when *L* < 0 Å, the average value of *F*_x_ is negative, whereas when *L* ≥ 0 Å, the average value of *F*_x_ becomes positive. Furthermore, with an increase in the numerical value of *L*, the average nanoindentation force *F*_x_ gradually increases. However, it was noticed that there are significant differences in the magnitude of *F*_x_ at symmetric positions (such as *L* = −100 Å and *L* = 100 Å), which is attributed to the anisotropy of monocrystalline copper. On the other hand, the average differences in *F*_y_ at various positions are relatively small, with lower numerical values. This phenomenon is attributed to the periodic boundary conditions. Additionally, it was observed that *F*_z_ is consistently greater than zero at different indentation positions, with the highest numerical value occurring at *L* = 0 Å, which is 503.3 nN. As *L* increases, the average nanoindentation force *F*_z_ decreases. However, the magnitude of the decrease at symmetrical positions (such as *L* = −100 Å and *L* = 100 Å) varies, similarly to the pattern observed for *F*_x_, and is also influenced by the anisotropy of monocrystalline copper.

**Figure 13:**
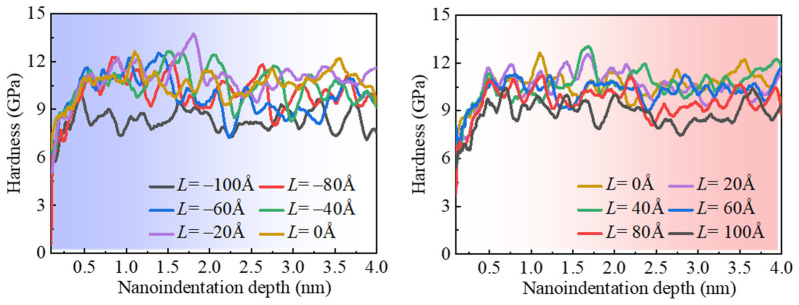
The variation in indentation hardness with indentation depth for different indentation positions.

Figure 13 shows the variation in nanoindentation hardness with respect to indentation depth under different indentation positions denoted as *L*. It can be observed that when the indentation depth is less than 0.5 nm, the nanoindentation hardness increases with increasing indentation depth. This is mainly because, in the initial stages of indentation with a shallow depth, the change in the contact area of the indentation is more pronounced within this depth range under the influence of a 5 nm radius diamond indenter. Conversely, it was observed that once the indentation depth exceeds 0.5 nm, the nanoindentation hardness essentially reaches a balanced state among different indentation positions.

**Figure 14:**
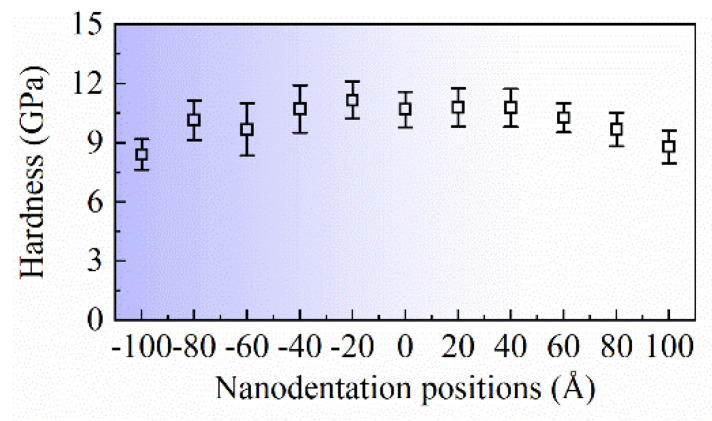
The variation in indentation hardness with respect to indentation position.

Figure 14 depicts the average nanoindentation hardness and corresponding variance at different indentation positions at a depth range of 1 nm to 3.5 nm. No statistical data are shown for the 0–1 nm range because the nanoindentation hardness did not stabilize within that range. It can be observed that there is a pronounced edge effect on the nanoindentation hardness on the (0 2 5) crystal plane of the monocrystalline copper specimen. It was observed that when *L* = −20 Å, the nanoindentation hardness reached its maximum value—approximately 7.8 GPa. As the indentation position approaches the boundary of the specimen, the nanoindentation hardness gradually decreases. Specifically, for *L* = −100 Å and *L* = 100 Å, the corresponding nanoindentation hardness values are 5.8 GPa and 6.2 GPa, respectively. Additionally, due to the inherent asymmetry of the (0 2 5) crystal orientation, the edge effect on nanoindentation hardness also exhibits significant anisotropic characteristics. The variation in nanoindentation hardness as *L* gradually decreases from 0 is more pronounced than the variation when *L* gradually increases from 0.

## 4. Conclusions

This work employed MD simulation methods to investigate the edge effects of nanoindentation on monocrystalline copper, with a specific focus on the anisotropic characteristics induced by the (0 2 5) crystal orientation. A detailed analysis was conducted to assess the impact of edge effects on material surface morphology, stress distribution, indentation force, and indentation hardness. The main conclusions are as follows:

(1) During the nanoindentation process of monocrystalline copper, significant surface sink-in and side squeeze-out phenomena were observed. The variation in indentation position led to differences in surface sink-in and side squeeze-out due to the activation of different [1 1 0] slip directions within the specimen. Specifically, surface sink-in in the specimen manifested as isosceles triangles with angles of 35° or 60°, while side squeeze-out exhibited differences in the distribution of isosceles triangles with a central region or right-angled triangles on both sides.
(2) Regarding the von Mises stress distribution, it was found that the primary activated sliding directions, namely *s*_1_ and *s*_4_, induced the propagation of von Mises stress in the *X*-*Z* plane. For −60 Å ≤ *L* ≤ 80 Å, von Mises stress propagated inward along the s1 sliding direction. As the indenter approached the edges of the specimen, the area of von Mises stress distribution gradually decreased due to the influence of the *s*_4_ sliding direction inside the specimen, leading to a weakening of the edge effect. In the *Y*-*Z* plane, von Mises stress was primarily concentrated near the indenter and did not propagate into the specimen’s interior, as there were no corresponding [1 1 0] slip directions in the *Y*-*Z* plane.
(3) Regarding the indentation force and hardness, it was observed that with increasing indentation depth, the fluctuation of the indentation force *F*_y_ became more pronounced. Simultaneously, when the indentation position approached the edges of the specimen, the unbalanced loading phenomenon of the indentation force *F*_x_ became more pronounced, while the indentation force *F*_z_ decreased. The nanoindentation hardness exhibited a significant edge effect on the (0 2 5) crystal plane of monocrystalline copper. The asymmetric (0 2 5) indentation plane of the monocrystalline copper specimen also resulted in pronounced anisotropic characteristics in the edge effect of the nanoindentation hardness. As the indentation position approached the edges of the specimen, the nanoindentation hardness gradually decreased.

## Figures and Tables

**Table 1:** Computational parameters of MD simulations.

Nanoindentation Parameters	Value
Specimen material	Monocrystalline copper
Specimen dimensions (nm^3^)	30 × 30 × 15
Loading velocity (m/s)	50
Nanoindentation depth (nm)	3.5
Potential function	EAM, Morse
Initial temperature (K)	293
Timestep (fs)	1

**Table 2:** The Morse parameters between different elements.

	*D* (eV)	*α*A − B (Å − 1)	*r*A-B (Å)
C-Cu	0.087	5.140	2.05

## Data Availability

The original contributions presented in this study are included in the article. Further inquiries can be directed to the corresponding authors.
